# Social skills training for drug users under treatment: a pilot study with follow-up

**DOI:** 10.1186/s41155-018-0109-9

**Published:** 2018-11-06

**Authors:** Jéssica Limberger, Ilana Andretta

**Affiliations:** Programa de Pós-Graduação em Psicologia Clínica, Universidade do Vale do Rio dos Sinos, (UNISINOS), Av. Unisinos, 950. Cristo Rei, São Leopoldo, RS CEP: 93020-190 Brazil

**Keywords:** Social skills training, Social skills, Substance-related disorders, Therapeutic communities

## Abstract

Drug abuse is associated with loss of social skills by drug users. The literature review revealed a strong need for social skills training as a strategy to assist in psychosocial rehabilitation. However, few studies to date have evaluated the results of social skills training in therapeutic communities (type of treatment often used for drug users). Therefore, the aim of the present study is to describe the results of a pilot study regarding the social skills training of drug users under treatment. This is a quasi-experimental pilot study, with pre- and post-tests and follow-up. The following assessment instruments were used for data collection: Questionnaire on Sociodemographic Data and Drug Abuse; Multidimensional Scale of Social Expression; Depression, Anxiety and Stress Scale, and The World Health Organization Quality of Life Assessment. The social skills training led to a significant increase in the skills of making refusals and expressing negative affect, quality of life (psychological domain), and a significant decrease in depressive symptoms and quality of life (environment domain). The high rate of intervention adherence (81.25%; *n* = 13) is indicative of the benefits from therapeutic community treatment and justifies the need for further empirical research, especially in terms of protocol development.

## Introduction

Social interactions are crucial in the human life cycle, i.e., both in family relationships and in the workplace; social skills are invaluable for quality of life. In this context, social skills are a set of behaviors expressed by a person in their environment, in which they will express their feelings, opinions, and rights in an adaptive manner, thereby decreasing the probability of future problems (Caballo, 1987).

Since the mid-1970s, social skills training (SST) has been used with the objective of developing specific social skill behaviors so that new behaviors can be integrated into an individual’s repertoire, by means of instructions, modeling, behavior tests, feedback, and reinforcement (Caballo, 2003; Del Prette & Del Prette, 2014). Since SST started being used, the scientific community has been discussing its effects on participants, especially the maintenance of social skills over time.

SST is frequently applied with an educational and preventive approach, on children, and with a therapeutic approach, as an ancillary strategy for treatment of psychiatric comorbidities (Del Prette & Del Prette, 2011). The benefits of SST have long been identified. A meta-analysis of SST with adults in psychiatric conditions identified improvements in their social skills repertoire which were maintained months after treatment, and showed that outpatient settings were the most adequate places for an intervention (Corrigan, 1991).

Particularly in the treatment of substance use disorder (SUD), SST was identified as a necessary component of treatment for drug users because these patients have deficits in particular social skills (SS) (Schneider, Limberger, & Andretta, 2016). Therapeutic communities (TCs) provide continuous care to users; hence, they are a much sought-after venue. The communities are characterized by constant social interaction, and interpersonal difficulties can hinder adhesion to the treatment (Staiger et al., 2014).

Severe symptomatology in SUD and the presence of psychiatric comorbidities often lead to treatment abandonment in therapeutic communities (Staiger et al., 2014). Thus, SST can be seen from the perspective of psychosocial rehabilitation, in order to handle losses resulting from the disorder and increase the potentialities of drug users in their social interactions, during the treatment and also afterwards (Schneider, Limberger, Novello, & Andretta, 2016). For these reasons, skills such as speaking with strangers, searching for a job, making new friends and developing relationships, and finding a support network are crucial in the development of a new lifestyle, a basic premise of SUD treatment.

Literature about SST for treatment of drug users is still lacking, according to a systematic review of the literature (Limberger, Trintin-Rodrigues, Hartman, & Andretta, 2017). This review was done on papers from the MEDLINE Complete, Scopus, IBECS, Index Psi, and LILACS databases and brings to light five studies that performed SST with drug users under treatment. Although literature on SST in SUD treatment is scarce, the main points revealed indicate that the subjects in the group which participated in SST significantly increased their assertive behavior between the pre-test and the post-test, when compared to the control group (Lin, Bon, Dickinson, & Blume, 1982). In the other studies, the results for SST were evaluated together with other interventions, and they show more patient engagement during the treatment (Tenhula, Bennett, & Kinnaman, 2009), significant efficacy in comparison to the habitual treatment, reduction of drug use, and increase in ability to perform daily activities (Bellack, Bennet, Gearon, Brown, & Yang, 2006), increased treatment adhesion, and reduction in the length of hospital stay and drug use (Petersen et al., 2007). Moreover, increased attention and decreased depressive symptomatology stood out as predictive factors in intervention efficacy (Teichner, Horner, & Harvey, 2001).

It is presumed that SUD is a maladaptive mode for individuals to handle interpersonal situations and stressor events (Caballo, 2003). On the other hand, symptoms of depression, anxiety, and stress can prevent individuals from expressing a socially skilled repertoire, thus negatively impacting their quality of life (Del Prette & Del Prette, 2011). In these terms, because of positive results achieved with SST such as improved social skills and increased treatment adhesion (Limberger et al., 2017) and despite the scarce literature on SST in SUD treatment, the review showed the need for evidence-based interventions to be developed and their effects to be assessed in the context of therapeutic communities. Therefore, the aim of the present study is to describe the results of a pilot study regarding the social skills training of drug users under treatment.

## Method

### Design

This is a quantitative, quasi-experimental pilot study with pre-test, post-test, and follow-up (Sampieri, Collado, & Lucio, 2013).

### Participants

**Fig. 1:**
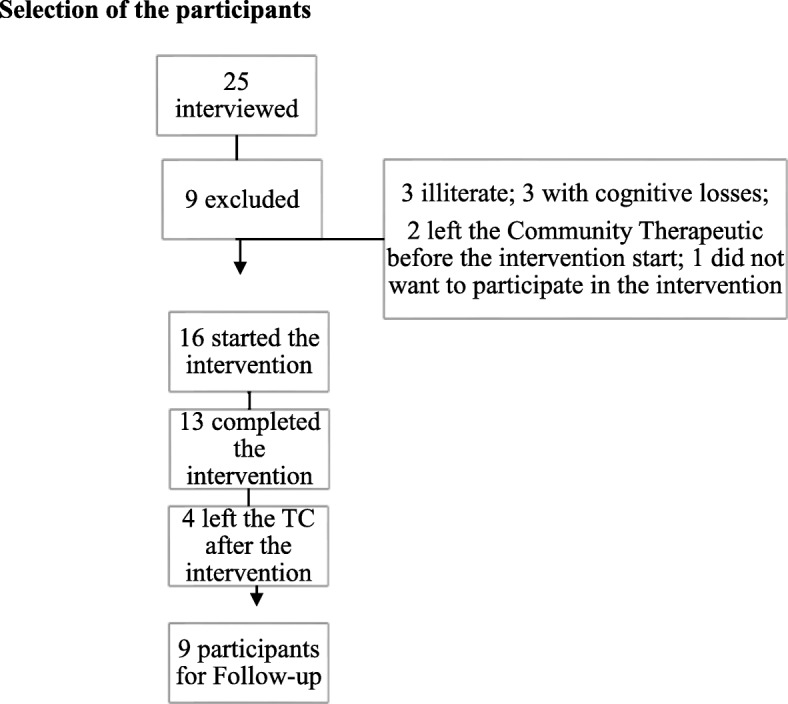
Flowchart of the selection of the participants—community therapeutic. Selection of the participants

In this study, 25 drug users were evaluated and 13 participants were included in the study, as shown in Fig. 1.

The 13 participants were males who had had been diagnosed with SUD and were under treatment in a therapeutic community (TC) in the northwest region of the state of Rio Grande do Sul (Brazil). Participants with the minimum of 75% frequency in SST were included (participating in at least six of the eight meetings). Participants who were illiterate (evaluated by self-report inventories) and/or had cognitive impairment (evaluated by Cognitive Screening with WAIS-III) were excluded from the study.

The participants were of a mean age of 39.38 years old (SD = 9.49). Educational attainment was categorized as follows (number of participants in brackets): some elementary school, no degree (4); elementary school (4); some high school, no degree (3); high school (1); and some college, no degree (1). Most of the participants were receiving treatment for crack abuse (61.5%; *n* = 8), followed by treatment for alcohol abuse (38.5%; *n* = 5).

### Assessment instruments

#### Questionnaire on Sociodemographic Data and Drug Abuse

This questionnaire was developed by the research group “Cognitive-Behavioral Interventions: Study and Research [*Intervenções Cognitivo-Comportamentais: Estudo e Pesquisa* - ICCEP]” and is aimed at assessing sociodemographic data, the criteria of the DSM-5 (American Psychiatric Association [APA], 2014) for diagnosis of SUD and the Brazilian Criteria of Economic Classification (BCEC), from the Associação Brasileira de Empresas de Pesquisa (ABEP, 2015).

#### Cognitive Screening with WAIS-III

This is a test for the exclusive use of psychologists, developed by Wechsler (1997) and adapted and standardized for Brazilian Portuguese by Nascimento (2004). Screening includes vocabulary and block design subtests and is aimed at evaluating cognitive impairment. The vocabulary subtest evaluates verbal comprehension of words presented to the examinee, who is supposed to define them orally. The block design subtest evaluates perceptual organization through a set of two-dimensional geometric patterns that the examinee must reproduce by using two-color cubes. Subtracting the weighted score of the vocabulary subtest from the weighted score of the block design test, the result is a difference of three points or more, which indicates cognitive impairment, according to Feldens, Silva, and Oliveira (2011). Cognitive impairment was an exclusion criterion in the present study.

#### Multidimensional Scale of Social Expression—Motor Part (EMES-M)

This scale was developed by Caballo (1987) and translated and adapted to Brazilian Portuguese by Pereira (2015). It is based on a 5-point Likert scale, which ranges from “Never or very rarely” to “Always or very often,” and it is intended to assess social skills in adults. The original version contains 65 items and 12 factors while the adapted version for Portuguese has 56 of the original items and eight factors (Pereira, 2015). In the present study, the omega values of the full scale were 0.678 in T1 (1 week before the intervention), 0.654 in T2 (1 week after the intervention), and 0.716 in T3 (a month after the intervention).

#### Depression, Anxiety and Stress Scale (DASS-21)

The DASS-21 was developed by Lovibond and Lovibond (1995), and adapted and validated for Brazilian Portuguese by Vignola and Tucci (2014). The scale has 34-point subscales, and each subscale is made up of seven items, which simultaneously assess the emotional states of depression, anxiety, and stress during the last week. The symptoms evaluated in the depression subscale are inertia, anhedonia, dysphoria, lack of interest/involvement, self-depreciation, devaluation of life, and discouragement. The anxiety subscale seeks to evaluate the following symptoms: excitation of the autonomic nervous system, musculoskeletal effects, situational anxiety, and subjective experiences of anxiety. The questions on the stress subscale assessment evaluate the following symptoms: difficulty in relaxing, nervous excitation, easy disruption/agitation, irritability/overreaction, and impatience. For calculation of the final score, the scores need to be multiplied by two. The classification of this assessment instrument corresponds to normal, medium, moderate, severe, and extremely severe. In the present study, the omega values were depression, 0.658 (T1), 0.661 (T2), 0.703 (T3); anxiety, 0.714 (T1), 0.658 (T2), 0.733 (T3); and stress, 0.722 (T1), 0.764 (T2), 0.736 (T3).

#### World Health Organization Quality of Life Assessment (WHOQOL-BREF)

This is an abbreviated version of WHOQOL-100, with 26 questions, focusing on the assessment of quality of life based on respondents’ perception. It is used by both healthy and clinical populations (World Health Organization (WHO), 1995). This assessment instrument has 24 facets that are grouped into four domains: physical health, psychological, social relationships, and environment. In addition, there are two questions about overall quality of life. Omega values obtained in this study were 0.843 in T1, 0.766 in T2, and 0.727 in T3.

### Ethical procedures

This study is part of a larger study entitled “Evaluation and Training in Social Skills in Chemical Dependents in Specialized Units,” approved by the Research Ethics Committee (Ethical Opinion Report No. 13.172) of the University of Vale do Rio dos Sinos (UNISINOS). After a letter of consent was sent by the Therapeutic Community Center, the patients were invited to participate in the research. An informed consent form was read out loud and explained individually to each participant to clarify that their participation was expected to be totally voluntary. The participants who agreed to join the research signed two copies of the form: one for the participant and one for the researcher.

### Data collection procedures

The assessment instruments were applied by psychologists and psychology students, members of ICCEP (a group for teaching and research on cognitive-behavioral interventions), with prior training. This training was conducted by professional psychologists with previous experience in the treatment of drug users and SST management. The trainees received a “Researcher Manual,” and undertook practical assessment instrument application activities within the group. Moreover, data collection was supervised by experienced professionals.

After qualification of the research group members, the first meeting was held in the Therapeutic Community Center. With the purpose of motivating participation, the researcher gave a lecture to all the therapeutic community users. In this lecture, practical activities were carried out on social skills, and the users were given explanations about the research and invited to participate. The research started that week.

The assessment instruments were applied individually in rooms at the therapeutic community facilities. Two meetings were held for the application of all the instruments, each lasting for approximately 2 hours. All the assessment instruments were read out loud to the participants to ensure they clearly understood the questions. It should be noted that in order to make the participants feel more comfortable when answering the questions, the researchers who applied the latter assessment instruments were not the same as those who applied the SST.

During the first research week, the illiterate participants were identified and the Cognitive Screening with WAIS-III was applied to identify the participants with cognitive loss. The participants who agreed with the inclusion criteria moved on to the next stage in the subsequent week when the following assessment instruments were applied: Questionnaire on Sociodemographic Data and Drug Abuse, Mini International Neuropsychiatric Interview (MINI), Multidimensional Scale of Social Expression—Motor Part (EMES-M), and Depression, Anxiety and Stress Scale (DASS-21).

After the application of the assessment instruments and allocation of marks, the research team gave feedback to the respondents; they emphasized those social skills which could be strengthened and those that required further practice. Next, the respondents were invited to participate in the social skills training, which started that week. The assessment instruments were applied at three different times: 1 week before the intervention (T1), 1 week after the intervention (T2), and a month after the intervention (T3).

### Intervention details

The intervention design was based on the review of literature and other references in the field of SST. The SST structure proposed by Lin et al. (1982) was selected: two 90-min weekly sessions for 4 weeks, in which the participants could choose the social skills they wished to develop.

The group format of the SST was decided on because it has benefits which tend to produce longer-lasting effects when compared to SST individual sessions (Argyle, Bryant, & Trower, 1974). All the sessions included the following elements:

1. Checking mood and diaphragmatic breathing
2. Homework review
3. Psychoeducation activity about the social skills to be learned
4. Social skills practice
5. Homework assignment
6. Feedback

The detailed description of each session is provided in Appendix.

The pilot study was conducted in a Therapeutic Community Center where the therapeutic program involved work and spiritual activities. During SST, the participants were encouraged to practice, with their peers, the skills they had learned in the intervention. The SST training was given by two psychologists with previous experience in the treatment of drug users. Both of them had participated in an SST training program to develop their SS. Subsequently, they conducted an SST training program with a non-clinical group and after that they conducted a pilot study under weekly supervision.

### Statistical analysis

The data were analyzed using the Statistical Package for Social Sciences—SPSS, version 20.0. Descriptive analysis covered frequencies, percentage, mean, and standard deviation of the sample. Inferential analysis was performed based on an analysis of repeated measures, when comparing the average of the variables (social skills, quality of life, symptoms of depression, anxiety, and stress) among the three assessment times (T1, T2, and T3). In that analysis, sphericity was assessed by Mauchly’s test and, in cases of sphericity violation, the Greenhouse-Geisser correction was used. In situations of high variability of the standard deviation (asymmetric distributions), Freedman’s non-parametric test was applied as a resource. Intervention effect size, in turn, was analyzed by Cohen’s *d*. For statistical decision criteria, the significance level of 5% was adopted (*p* value < 0.05). For the assessment instruments, a decision was made to calculate the omega instead of the alpha value, because according to Crutzen and Peters (2017), alpha is an inadequate estimate for the validity and reliability of a scale. McNeish (2017) points out that alternative measures, such as omega, present greater reliability compared to Cronbach’s alpha.

## Results

Thirteen drug users participated in the intervention in a treatment in a Therapeutic Community Center in the northwest region of the state of Rio Grande do Sul (Brazil). They account for the majority of the 16 patients (81.25%; *n* = 13) who had started the training and who had attended at least 75% of the sessions.

The social skills chosen by the participants on the first day of intervention were starting and keeping a conversation going, expressing positive affect, speaking in public, acknowledging compliments, making refusals, and defending rights. Such skills were practiced in this exact order in order to enable the participants to start working on the skills that they had considered to be the easiest and, thus, to increase their self-efficacy as regards their performance, according to Del Prette and Del Prette (2011).

After assessment of social skills’ quality of life and symptoms of depression, anxiety, and stress, before and after the intervention (T1 vs. T2), of the 13 participants who concluded the SST, only the refusal skill showed a significant increase (*t* = 2.808; *p* = 0.016). Therefore, as shown in Fig. 1, after evaluation of the participants who completed the follow-up (*n* = 9), statistically significant differences were detected among the three assessment times (T1, T2, and T3) in the social skills of making refusals (*χ*^2^_Friedman (2)_ = 4.226; *p* = 0.042) and expressing negative affect (*χ*^2^_Friedman (2)_ = 2.294; *p* = 0.032), depressive symptoms (*χ*^2^_Friedman (2)_ = 1.697; *p* = 0.028), and quality of life: psychological (*F*_(2;16)_ = 14.202; *p* = 0.001) and environment (*χ*^2^_Friedman (2)_ = 5.886; *p* = 0.043) domains. The social skill of making refusals decreased at 1 week after the intervention and increased again at 1 month after the intervention. Consequently, the skill of expressing negative affect increased significantly at 1 week after the intervention and continued to increase at 1 month after the intervention. Such increase was also seen for quality of life (psychological domain). In the environment domain of the quality of life scale, there was a significant decrease at 1 week after the intervention and then an increase after the first month. Finally, there was also a decrease in depressive symptoms at 1 week after the intervention, as well as after 1 month. As regards the effect size, there was a strong effect on quality of life (psychological domain), followed by an average effect on expressing negative affect and depression symptoms, as well as a weak effect on the variables of refusal and quality of life (environment domain), as shown in Table 1.

**Table 1:** Results of the intervention on social skills, depression, anxiety, stress, and quality of life

Variables	T1 Mean (SD)	T2 Mean (SD)	T3 Mean (SD)	*N*	Test statistic	*p*	*d* (T1 vs T3)
Making refusals^1^	13.444a (4.447)	10.000b (5.000)	13.777a (3.800)	9	*χ* ^2^_Friedman (2)_ 6.353	0.042*	0.080
Expressing Negative Affect^1^	19.333b (4.000)	20.777b (6.418)	22.444a (4.693)	9	*χ* ^2^_Friedman (2)_ 2.294	0.032*	0.71
Starting and keeping a conversation^1^	25.111a (7.321)	27.222a (8.318)	28.222a (9.175)		*χ* ^2^_Friedman (2)_ 0.667	0.717	NA
Acknowledging compliments^2^	6.888a (1.536)	7.444a (2.603)	7.333a (2.449)		F_(2;16)_ = 0.161	0.852	NA
Speaking in public^1^	6.777a (3.800)	6.555a (4.333)	8.888a (3.822)		*χ* ^2^_Friedman (2)_ 2.552	0.279	NA
Expressing Positive Affect^1^	26.555a 6.857	26.444a 9.056	26.444a 5.918		*χ* ^2^_Friedman (2)_ 1.235	0.539	NA
Expressing disagreement^2^	14.333a (4.924)	15.777a (4.994)	15.666a (5.220)		F_(2;16)_ = 0.679	0.521	NA
Defending rights^1^	14.111a (5.085)	15.666a (6.224)	17.555a (6.424)		*χ* ^2^_Friedman (2)_ 3.486	0.175	NA
Depression symptoms^1^	5.222a (4.116)	3.444b (3.643)	2.000a (4.242)	9	*χ* ^2^_Friedman (2)_ 1.697	0.028*	0.770
Anxiety Symptoms^1^	3.555a (2.743)	3.222a (2.990)	1.777a (1.715)	9	*χ* ^2^_Friedman (2)_ 5.154	0.076	NA
Stress symptoms^1^	5.555a (3.711)	3.666a (2.449)	2.666a (3.082)	9	*χ* ^2^_Friedman (2)_ 3.563	0.168	NA
Quality of life (psychological)^2^	3.274a (0.646)	4.015b (0.652)	4.255b (0.251)	9	*F*_(2;16)_ = 14.202	0.001**	2.189
Quality of life (environment)^1^	78.388a (6.913)	74.222b (2.181)	75.555b (5.433)	9	*χ*^2^_Friedman (2)_ 5.886	0.043*	− 0.458
Quality of life (physical)^2^	3.760a (0.753)	3.950a (0.927)	4.122a (0.793)	9	*F*_(2;16)_ = 1.307	0.298	NA
Quality of life (social)^2^	3.106a (0.833)	3.588a (0.722)	3.848a (0.853)	9	*F*_(2;16)_ = 2.283	0.134	NA

a/b: notation used in multiple comparisons to identify which means differ significantly, where means followed by the same letters (on the line) do not differ in a significance of 5%

*NA* not applicable

*p at the level of 0.05 **p at the level of 0.05

^1^Variables analyzed by the Friedman test

^2^Analysis of variance *F*-Snedecor

## Discussion

A pilot study makes it possible to anticipate problems and adapt procedures (Viechtbauer et al., 2015). Regarding problems, there was a decrease in the number of participants between the post-test and the follow-up, suggesting motivational strategies ought to be included. Just like the importance of awarding a certificate post-test, offering something that represents the conclusion of one more step, such as taking a group photograph, might motivate participants to continue the treatment and participate in the follow-up.

Among the findings from the pilot study, it was found that excluding illiterates from the study is unnecessary because all the assessment instruments were read out loud to the participants. In addition, in the social skills training, illiterate patients are also able to participate because the activities are practical. This means all the participants of the Therapeutic Community Center can be invited to participate in the intervention.

This pilot study showed the possibilities of SST for treatment of SUD, a field of study underexplored in the literature (Limberger et al., 2017). The development of social skills is one of the essential elements in relapse prevention (Ferrari, Smeraldi, Bottero, & Politi, 2014). The data of this pilot study indicated possible consequences of the intervention, e.g., a significant increase in some of the study variables, namely, social skills for making refusals and expressing negative affect, quality of life (psychological domain), and a significant decrease in symptoms of depression and quality of life (environment domain).

Refusal skills, including refusal of requests, confirmed via the systematic review of literature, have been practiced in interventions with drug users, as well as the development of drug refusal skills (Limberger et al., 2017). The skill of making refusals is also related to expressing negative affect, which showed a significant increase from T2 to T3, which is indicative that such skill requires a longer maturation time to be developed. It should be noted that practice is crucial for the improvement of social skills (Caballo, 2003).

The participants’ satisfaction with the intervention may have been a contributing factor for the high rate of adhesion to the training program. It was also found that the participants, by choosing which SS they wished to develop, feel more motivated to engage in the proposed activities. This motivation also stood out in the study by Lin et al. (1982), which highlighted the importance of participants’ choice of the SS they wish to practice in SST.

The experience of positive feelings, combined with enthusiasm for performing the activities and realizing a meaning in life, is a typical behavior of a decrease in depressive symptoms, as found in the present study. These behaviors may also have increased the participants’ adhesion to the intervention. Similarly, the decrease in depressive symptomatology was one of the predictive factors in the efficacy of the intervention in an American study with 63 drug users under treatment (Teichner et al., 2001). In the Brazilian scenario, participants who presented severe symptoms of depression had higher probability of deficits in social skills, according to the study with 519 crack users (Horta et al., 2016). These data suggest that a previous screening of participants, in order to identify those who have depressive symptomatology, may enable the offer of a specialized treatment, based on the individual needs of each patient.

The significant increase in quality of life, in the psychological domain, indicates that the participants are very satisfied with themselves and realize that their life has a meaning, and hence they want to enjoy it. As SST is an experience, when individuals practice new SS during the intervention, they may realize that they are more satisfied with themselves, instead of criticizing and avoiding those situations (Andretta, Limberger, & Schneider, 2016; Caballo, 2003).

Another noteworthy point is the decrease in the perception of quality of life in the environment domain. This result is indicative of individuals’ perception of the environment where they live, their financial resources, their access to information and services, and their physical environment (pollution, noise, etc.) (Organização Mundial da Saúde, 1995). After awareness that the environment is still the same (the participants remained in the TC center) but their perception had changed between one assessment time and another, it can be hypothesized that the participants were feeling more comfortable about evaluating and expressing dissatisfaction with the environment where they live, and hence they were more critical and more aware of their rights.

The limitations of the study also need to be taken into consideration so that the results can be understood more clearly. One example is the measurement of social skills using a self-report instrument. For this purpose, we suggest the inclusion of other instruments which can assess social skills in situations that are more similar to those experienced by the participants at the Therapeutic Community Center. Instruments such as grading on a scale about interpersonal situations experienced by the participants during the treatment, as well as a video-recorded semi-structured test of extensive interaction, would make the training situations resemble real situations experienced by the participants more accurately (Caballo, 2003).

Another limit of the study is that the sample is composed only of males. A study with 127 participants (Andretta et al., 2016) demonstrated there were differences between social skills of male and female crack users, where males presented greater difficulties. It should be stressed that social skills are performed according to their cultural context of origin, indicating that both the treatment of female drug users and social skills training need to consider the cultural specificities of female roles, rather than just replicate studies with males (Limberger, Schneider, & Andretta, 2015).

These results need to be analyzed carefully, particularly because this is a pilot study which, by default, is conducted with a small sample size (Viechtbauer et al., 2015). The small number of participants threatens external validity, because the data cannot be generalized. The effects are speculative because of inaccurate parameter estimates. Therefore, further research is needed on this type of intervention because a higher number of participants would help to substantiate the statistical analysis, along with a more elaborate protocol description.

## Conclusion

In the field of SST, a pilot study presents an advancement as it describes and evaluates SST applicability in SUD treatment. In the case of a group intervention, the participants observe the application of social skills in several ways, thereby expanding their behavioral repertoire when they practice such skills in a group. In addition, the group format also enhances the dynamics of the therapeutic community by enabling a larger number of users to benefit from the intervention.

The high rate of adhesion of participants to the intervention proves its potential in the therapeutic community because it may increase adhesion to the treatment as a whole as the practice of social skills helps to improve interpersonal relationships and quality of life. In fact, the SST was performed in a wider treatment context—the Therapeutic Community Center. In doing so, SST is a complementary strategy to the treatment of SUD without replacing other interventions such as Motivational Interviewing and Cognitive Behavioral Therapy.

The importance of offering previous social skills training to the professionals who will conduct SST is also paramount because modeling is a key factor in the practice of social skills. Moreover, the methodological decision for one team to apply the assessment instruments and another to conduct the intervention is very valid because it avoids the risk of participants giving biased answers.

## Appendix

**Table 2:** Description of sessions and procedures

Session and objective	Procedures
Session 1—Presentation Objective: To introduce the SST structure, motivating participants to continue to participate in the intervention	1) The participants introduce themselves and report how they are feeling. 2) They create their own name tag. The meetings are scheduled (dates and shifts); the meeting structure is explained; a poster listing the six steps of each session is placed in the middle of the circle formed by the group; explanations are given about attendance—they will receive a certificate of participation if they attend the minimum of 75% of the meetings and receive a stamp at each meeting they attend on a list visible to everyone. 3) Six social skills are selected to be worked on. 4) Exercise on Basic Human Rights, according to Caballo (2003). 5) Homework: choose one of the rights from the list that you deem most important for you at this moment 6) Feedback: What did you think about the meeting? How did you feel? What do you suggest for the next meeting?
Session 2—Starting and keeping a conversation going Objective: To understand the relevance of the skill *starting and keeping a conversation going* in different contexts and then practice it.	1) Mood check and diaphragmatic breathing. Explanations are given about the importance of breathing for well-being and decreasing anxiety; practice exercise by means of guided instruction. 2) Homework review. Positive reinforcement is offered to those who finished the task by showing the importance of SS practice. An imagination activity is performed to exercise the basic human right of choice. 3) Psychoeducation activity about the social skills to be learned: *starting and keeping a conversation going* Understanding verbal and non-verbal signals of a person’s interest in starting a conversation, types of question that create other conversational topics. 4) Social skills practice Bus activity: in pairs, each participant starts and keeps a conversation going, as if they were on a bus, until they hear a sound signal that notifies arrival at the destination, when they should say goodbye and speak to another pair. 5) Homework assignment: start and keep a conversation going with a colleague from the Therapeutic Community Center 6) Feedback.
Session 3—Expressing positive affect Objective: To understand the importance of expressing positive feelings to strengthen relationships, and practice such a skill.	1) Mood check and diaphragmatic breathing. Moment to talk about the previous week and see if the participants have some important information to share with the group 2) Homework review Emphasis on the need for SS practice to learn and develop it further 3) Psychoeducation about the social skill to be learned: expressing positive feelings Relation between the expression of positive feelings and the proximity between people, support network expansion 4) Social skill practice Empty Chair Exercise: an empty chair is placed in the middle of the group and each participant goes to it, as if there were a person to express positive feelings to there. 5) Homework attribution: Give a compliment to a colleague 6) Feedback
Session 4—Speaking in public Objective: Develop strategies to speak in public and practice such a skill.	1) Mood check and diaphragmatic breathing 2) Homework review Discuss how each participant felt giving a compliment: ease and difficulty 3) Psychoeducation activity about the social skill to be learned: *speaking in public* Strategies that can help (look for those who are paying attention, get to know the public and the place, etc.) 4) Social skill practice Empty Screen Exercise: each person has 1 minute to speak about objects that are randomly taken from a bag Discussion on how they felt while doing the exercise and how they can deal with unexpected events. Importance of daily diaphragmatic breathing to decrease anxiety while speaking in public. 5) Homework assignment Practice diaphragmatic breathing and make a note of how many times they did that during the week. 6) Feedback
Session 5—Acknowledging compliments Objective: To acknowledge assertive compliments and acknowledge their own qualities.	1) Mood check and diaphragmatic breathing Discuss how they are feeling and how their week was 2) Homework review Discuss how they felt after doing the daily diaphragmatic breathing and if they felt better after respiration. 3) Psychoeducation activity about the social skill to be learned: *Acknowledging compliments* Understanding the importance of compliments in different contexts, as well as recognizing their skills 4) Social Skill practice In a circle, each participant practices the compliment technique and asks a question so that the other person can acknowledge the compliment more easily. 5) Homework assignment Give a compliment to some colleagues and add a question about the theme of the compliment. 6) Feedback.
Session 6—Making refusals Objective: To recognize the right of refusal and practice it.	1) Mood check and diaphragmatic breathing 2) Homework review Discuss a peer’s reaction after receiving a compliment. 3) Psychoeducation activity about the social skill to be learned – Making refusals Teach strategies such as “Scratched Disc” (Caballo, 2003), asking for more time to think, asking for clarifications, being brief, etc. 4) Social skill practice Based on situations that the group considers to be more difficult to refuse, each pair will act out how they can assertively say no in that situation. 5) Homework assignment Refusing a command that you do not wish to obey 6) Feedback.
Session 7—Defending rights Objective: To practice the skill of defending rights, while clearly understanding respect for oneself and for others	1) Mood check and diaphragmatic breathing 2) Homework review Discuss feelings arising from making refusals; the importance of being aware of rights that must be respected by oneself and by others. 3) Psychoeducation activity about the social skill to be learned – defending rights. Context of defense of rights, transparency about one’s own rights 4) Social skill practice Practice of product exchange: each participant acts out as if they were in a store. Discussion about alternatives and ways to defend their rights. 5) Homework assignment Choosing one of the skills that have been learned during the training and practice it. 6) Feedback.
Session 8—Conclusion Objective: To think about the social skills that have been learned, and create confrontation cards to be used on a daily basis.	1) Mood check and diaphragmatic breathing Discussion about the previous meeting and the importance for the group to continue practicing social skills 2) Homework review 3) Creation of confrontation cards Using sheets of paper with the names of each social skill, review ways to exercise those social skills in several situations. Next, after the group discussion, each participant creates a confrontation card for each social skill, with hints and strategies for development of social skills. 4) Feedback The participants assess the SST training and make suggestions about it.

## Abbreviations

SS: Social skills
SST: Social skills training
SUD: Substance use disorder

## References

[CR1] American Psychiatric Association [APA] (2014). Manual Diagnóstico e Estatístico de Transtornos Mentais.

[CR2] Andretta I, Limberger J, Schneider JA (2016). Social skills in crack users: Differences between men and women. Psicologia: Reflexão e Crítica.

[CR3] Argyle M, Bryant B, Trower P (1974). Social skills training and psychotherapy: A comparative study. Psychological Medicine.

[CR4] Associação Brasileira de Empresas de Pesquisa [ABEP]. (2015). Brazilian Economic Classification Criterial. Retrieved from http://www.abep.org/criterio-brasil.

[CR5] Bellack AS, Bennet ME, Gearon JS, Brown CH, Yang Y (2006). A randomized clinical trial of a new behavioral treatment for drug abuse in people with severe and persistent mental illness. Archives of General Psychiatry.

[CR6] Caballo VE (1987). Evaluación de las habilidades sociales: una estrategia multimodal.

[CR7] Caballo VE (2003). Manual de avaliação e treinamento das habilidades sociais.

[CR8] Corrigan PW (1991). Social skills training in adult psychiatric populations: A meta-analyis. Journal of Behavior Therapy and Experimental Psychiatry.

[CR9] Crutzen R, Peters G-JY (2017). Scale quality: Alpha is an inadequate estimate and factor-analytic evidence is needed first of all. Health Psychology Review.

[CR10] Del Prette ZAP, Del Prette A (2011). Habilidades sociais: intervenções efetivas em grupo.

[CR11] Del Prette ZAP, Del Prette A (2014). Psicologia das relações interpessoais: vivências para o trabalho em grupo.

[CR12] Feldens, A. C. M., Silva, J. G. D., & Oliveira, M. D. S. (2011). Assessment of executive function in alcoholics. *Cadernos de Saúde Coletiva*, *19*, 164–171 Retrieved from http://www.cadernos.iesc.ufrj.br/cadernos/images/csc/2011_2/artigos/csc_v19n2_164-171.pdf.

[CR13] Ferrari V, Smeraldi E, Bottero G, Politi E (2014). Addiction and empathy: A preliminary analysis. Neurological Sciences.

[CR14] Horta, R. L., Schäfer, J. L., Coelho, L. R. M., Rodrigues, V. S., Oliveira, M. S., & Teixeira, V. A. (2016). Conditions associated with impaired social skills in a convenience sample of crack users. *Cadernos de Saúde Pública*, *32*(4). 10.1590/0102-311X00010715. 10.1590/0102-311X00010715 27096296

[CR15] Limberger Jéssica, Schneider Jaluza Aimèe, Andretta Ilana (2015). Especificidades do tratamento de mulheres usuárias de crack: interface com direitos humanos. Psicologia em Pesquisa.

[CR16] Limberger, J., Trintin-Rodrigues, V., Hartman, B., & Andretta, I. (2017). Social skills training for drug users: systematic literature review. *Contextos Clínicos*, *10*(1), 99–109. 10.4013/ctc.2017.101.08.

[CR17] Lin Tien-Teh, Bon Shelly, Dickinson Joan, Blume Cathy (1982). Systematic Development and Evaluation of a Social Skills Training Program for Chemical Abusers. International Journal of the Addictions.

[CR18] Lovibond PF, Lovibond SH (1995). The structure of negative emotional states: Comparison of the Depression Anxiety Stress Scales (DASS) with the Beck depression and anxiety inventories. Behaviour Research and Therapy.

[CR19] McNeish Daniel (2018). Thanks coefficient alpha, we’ll take it from here. Psychological Methods.

[CR20] Nascimento E (2004). Adaptação e padronização brasileira da Escala de Inteligência Wechsler para Adultos.

[CR21] Pereira AS (2015). Avaliação das habilidades sociais e suas relações com fatores de risco e proteção em jovens adultos brasileiros.

[CR22] Petersen L, Jeppesen P, Thorup A, Ohlenschlaeger J, Kraup G, Ostergard T (2007). Substance abuse and first-episode schizophrenia-spectrum disorders. The danish OPUS trial. Early Intervention in Psychiatry.

[CR23] Sampieri RH, Collado CF, Lucio MPB (2013). Metodologia de Pesquisa.

[CR24] Schneider, J. A., Limberger, J., & Andretta, I. (2016). Social Skills and Drugs: Systematic Review of National and International Scientific Production. *Avances en Psicología Latinoamericana*, *34*(2), 339–350. 10.12804/apl34.2.2016.08.

[CR25] Schneider, J. A., Limberger, J., Novello, B., & Andretta, I. (2016). The role of psychosocial rehabilitation in crack user treatment. *Aletheia*, *49*, 35–47 Retrieved from http://www.periodicos.ulbra.br/index.php/aletheia/article/view/3596/2656.

[CR26] Staiger PK, Kyrios M, Williams JS, Kambouropoulos N, Howard A, Gruenert S (2014). Improving the retention rate for residential treatment of substance abuse by sequential intervention for social anxiety. BMC Psychiatry.

[CR27] Teichner G, Horner MD, Harvey RT (2001). Neuropsychological predictors of the attainment of treatment objectives in substance abuse patients. International Journal of Neuroscience.

[CR28] Tenhula WN, Bennett ME, Kinnaman JES (2009). Behavioral treatment of substance abuse in schizophrenia. Journal of Clinical Psychology.

[CR29] Viechtbauer W, Smits L, Kotz D, Budé L, Spigt M, Serroyen J, Crutzen R (2015). A simple formula for the calculation of sample size in pilot studies. Journal of Clinical Epidemiology.

[CR30] Vignola RC, Tucci AM (2014). Adaptation and validation of the depression, anxiety and stress scale (DASS) to Brazilian Portuguese. Journal of Affective Disorders.

[CR31] Wechsler D (1997). Weschsler Adult Intelligence Scale-III.

[CR32] World Health Organization (WHO). (1995). The World Health Organization quality of life assessment (WHOQOL): Position paper from the World Health Organization. Social Science & Medicine, *41*(10):1403-1409. https://doi.org/10.1016/0277-9536(95)00112-K . 10.1016/0277-9536(95)00112-k 8560308

